# Non-Sentinel Lymph Node Metastasis Prediction in Breast Cancer with Metastatic Sentinel Lymph Node: Impact of Molecular Subtypes Classification

**DOI:** 10.1371/journal.pone.0047390

**Published:** 2012-10-09

**Authors:** Fabien Reyal, Catherine Belichard, Roman Rouzier, Emmanuel de Gournay, Claire Senechal, Francois-Clement Bidard, Jean-Yves Pierga, Paul Cottu, Florence Lerebours, Youlia Kirova, Jean-Guillaume Feron, Virginie Fourchotte, Anne Vincent-Salomon, Jean-Marc Guinebretiere, Brigitte Sigal-Zafrani, Xavier Sastre-Garau, Yann De Rycke, Charles Coutant

**Affiliations:** 1 Department of Surgery, Institut Curie, Paris, France; 2 Department of Medical Oncology, Institut Curie, Paris, France; 3 Department of Radiation Oncology, Institut Curie, Paris, France; 4 Department of Tumour Biology, Institut Curie, Paris, France; 5 Department of Biostatistic, Institut Curie, Paris, France; 6 Department of Surgery, Centre Georges-Francois Leclerc, Dijon, France; Ospedale Pediatrico Bambino Gesu', Italy

## Abstract

**Introduction:**

To decipher the interaction between the molecular subtype classification and the probability of a non-sentinel node metastasis in breast cancer patients with a metastatic sentinel lymph-node, we applied two validated predictors (Tenon Score and MSKCC Nomogram) on two large independent datasets.

**Materials and Methods:**

Our datasets consisted of 656 and 574 early-stage breast cancer patients with a metastatic sentinel lymph-node biopsy treated at first by surgery. We applied both predictors on the whole dataset and on each molecular immune-phenotype subgroups. The performances of the two predictors were analyzed in terms of discrimination and calibration. Probability of non-sentinel lymph node metastasis was detailed for each molecular subtype.

**Results:**

Similar results were obtained with both predictors. We showed that the performance in terms of discrimination was as expected in ER Positive *HER2* negative subgroup in both datasets (MSKCC AUC Dataset 1 = 0.73 [0.69–0.78], MSKCC AUC Dataset 2 = 0.71 (0.65–0.76), Tenon Score AUC Dataset 1 = 0.7 (0.65–0.75), Tenon Score AUC Dataset 2 = 0.72 (0.66–0.76)). Probability of non-sentinel node metastatic involvement was slightly under-estimated. Contradictory results were obtained in other subgroups (ER negative *HER2* negative, *HER2* positive subgroups) in both datasets probably due to a small sample size issue. We showed that merging the two datasets shifted the performance close to the ER positive *HER2* negative subgroup.

**Discussion:**

We showed that validated predictors like the Tenon Score or the MSKCC nomogram built on heterogeneous population of breast cancer performed equally on the different subgroups analyzed. Our present study re-enforce the idea that performing subgroup analysis of such predictors within less than 200 samples subgroup is at major risk of misleading conclusions.

## Introduction

Sentinel lymph node (SN) biopsy can accurately stage the axilla in early breast cancer, and it causes less morbidity than axillary lymph node dissection (ALND) [13], [17], [21]. It remains to be determined whether ALND is always required for women with positive SNs on final histology, given that 40% to 70% of these patients have no metastatic non-sentinel lymph nodes (non-SNs) [2], [3], [8], [15]. The likelihood of non-SN metastasis depends on several factors, such as histologic primary tumour size, the size of SN metastasis, the number of positive SNs, the ratio of positive SNs to all removed SNs, and the extracapsular extension status of the positive SNs [1], [3], [6], [8], [15], [19], [20], [22]. However, none of these characteristics by themselves can identify a subset of patients for whom ALND is unnecessary. Coutant et al published a prospective study of 9 multivariate models designed to predict non-sentinel lymph node status in patients with sentinel node metastasis [4]. They showed that the different models do not perform equally and that MSKCC nomogram and Tenon Score were the most accurate to determine non-sentinel nodes status.

**Table 1:** Clinical and pathological features Dataset 1.

Clinical and pathological features of 654 breast cancer samples with a positive sentinel node biopsy (Dataset 1)						
	All Samples	ERpos HER2neg	ERneg HER2neg	ERpos HER2pos	ERneg HER2pos	pvalue
**Samples**	654	573 (88%)	22 (3%)	32 (5%)	27 (4%)	
**Age at diagnosis**						
Median (Range)	57 (27–88)	58 (31–88)	56 (36–74)	55 (27–78)	54 (27–74)	NS
**pT**						
T1	516 (79%)	455 (79%)	18 (86%)	24 (75%)	19 (66%)	NS
T2	132 (20%)	112 (20%)	9 (14%)	8 (25%)	3 (33%)	
T3	6 (1%)	6 (1%)	0	0	0	
Median (Range)	15 (0–100)	15 (0–100)	15 (2–40)	15 (0–100)	17 (5–37)	NS
**Histological type**						
Ductal	554 (85%)	483 (84%)	18 (82%)	27 (84%)	26 (93%)	9e-05
Lobular	87 (13%)	82 (14%)	1 (5%)	4 (12%)	0 (0%)	
Other	13 (2%)	8 (2%)	3 (13%)	1 (4%)	1 (7%)	
**Elston Ellis**						
I	154 (23%)	152 (26%)	1 (4%)	1 (3%)	0 (0%)	2e-16
II	358 (55%)	323 (56%)	6 (27%)	19 (59%)	10 (37%)	
III	142 (22%)	98 (17%)	15 (68%)	12 (38%)	17 (63%)	
**Lymphovascular space involvment**						
Positive	237 (36%)	197 (34%)	8 (36%)	15 (47%)	17 (60%)	0.02
**Multifocal**						
Positive	129 (20%)	111 (19%)	0 (0%)	10 (31%)	8 (29%)	0.02
**Sentinel Nodes Removed**						
Median (Range)	2 (1–12)	2 (1–12)	3 (1–7)	2 (1–7)	2 (1–9)	NS
**Number of Metastatic Sentinel Nodes**						
1	533 (81%)	461 (80%)	22 (100%)	28 (88%)	22 (79%)	NS
2	93 (14%)	90 (16%)		2 (6%)	1 (7%)	
3	23 (4%)	18 (3%)		2 (6%)	3 (11%)	
>3	5 (1%)	4 (1%)			1 (3%)	
**Ratio Number of Positive Nodes/Number of Examined Sentinel Nodes**						
Median (Range)	0.5 (0.1–1)	0.5 (0.1–1)	0.3 (0.15–1)	0.5 (0.14–1)	0.5 (0.1–1)	NS
**Diagnostic**						
IHC	103 (16%)	97 (17%)	1 (5%)	4 (13%)	1 (4%)	NS
Micro	172 (26%)	149 (26%)	5 (23%)	10 (31%)	8 (29%)	
Macro	379 (58%)	327 (57%)	16 (73%)	18 (56%)	18 (68%)	
**Metastatic non sentinel axillary lymph node**						
Positive	179 (27%)	154 (27%)	2 (9%)	13 (41%)	10 (39%)	0.03
**Number of metastatic non sentinel axillary lymph node**						
0	475 (73%)	419 (73%)	20 (91%)	19 (59%)	17 (61%)	NS
1	87 (13%)	75 (13%)	2 (9%)	6 (19%)	4 (14%)	
2	32 (5%)	29 (5%)	0	2 (6%)	1 (7%)	
3	20 (3%)	16 (3%)	0	2 (6%)	2 (7%)	
>3	40 (6%)	34 (6%)	0	3 (9%)	3 (10%)	

We recently published a study showing the strong interaction between the breast cancer molecular subtypes classification as determined by estrogen receptor (ER) and *HER2* immuno-staining (ER positive HER2 negative | ER negative HER2 negative | ER negative HER2 positive | ER positive HER2 positive) and the risk of metastatic axillary sentinel lymph node [16]. We showed for each molecular subtype a specific correlation pattern between the tumour size and the probability of a positive sentinel node biopsy. Using tumour size, lympho-vascular invasion, molecular subtypes classification and age at diagnosis, we designed a multivariate logistic regression model to determine the probability of having a positive sentinel node biopsy. These results suggest that the axillary lymph node metastasis process is predominantly correlated to intrinsic biological properties in the ER negative *HER2* negative breast cancer subgroup whereas stochastic events, tumour size, growth rate and lympho-vascular invasion are the main determinants in the ER positive or *HER2* positive breast cancer subgroups. It is however unknown if this finding is exportable for non-sentinel lymph node. Notably, it is unknown if the Tenon Score and the MSKCC nomogram perform equally in the different breast cancer subgroups as defined by the immune-phenotype molecular subtypes classification.

**Table 2:** Clinical and pathological features Dataset 2.

Clinical and pathological features of 574 breast cancer samples with a positive sentinel node biopsy (Dataset 2).						
	All Samples	ERpos HER2neg	ERneg HER2neg	ERpos HER2pos	ERneg HER2pos	pvalue
**Samples**	574	480 (84%)	45 (8%)	32 (6%)	17 (3%)	
**Age at diagnosis**						
Median (Range)	57 (29–84)	57 (31–84)	58 (29–78)	54 (37–78)	52 (37–76)	NS
**TSize**						
Median (Range)	15 (1–60)	15 (1–60)	15 (4–50)	15 (8–45)	14 (2–24)	NS
**pTSize**						
pT1	467 (81%)	390 (81%)	35 (78%)	26 (81%)	16 (94%)	NS
pT2	103 (18%)	86 (18%)	10 (22%)	6 (19%)	1 (6%)	
pT3	3 (0.5%)	3 (1%)	0	0	0	
**Histological type**						
Ductal	509 (89%)	419 (87%)	43 (95%)	31 (97%)	16 (95%)	NS
Lobular	65 (11%)	61 (13%)	2 (5%)	1 (3%)	1 (5%)	
**Elston Ellis**						
I	198 (34%)	175 (36%)	10 (22%)	9 (28%)	4 (23%)	4e-6
II	283 (49%)	246 (51%)	19 (42%)	13 (40%)	5 (29%)	
III	88 (15%)	55 (11%)	16 (35%)	10 (31%)	7 (41%)	
**Lymphovascular space involvement**						
Positive	231 (40%)	184 (38%)	21 (46%)	18 (56%)	8 (47%)	NS
**Sentinel Nodes Removed**						
Median (Range)	1 (1–5)	1 (1–5)	1 (1–2)	1 (1–4)	1 (1–3)	NS
**Number of Metastatic Sentinel Nodes**						
1	519 (90%)	433 (90%)	43 (95%)	28 (87%)	15 (88%)	6.8e-05
2	48 (8%)	43 (9%)	2 (5%)	2 (6%)	1 (6%)	
3	3 (0.5%)	3 (0.6%)	0	0	0	
>3	4 (0.7%)	1 (0.2%)	0	2 (6%)	1 (6%)	
**Ratio Number of Positive Nodes/Number of Examined Sentinel Nodes**						
Median (Range)	1 (0–2)	1 (0.5–1)	1 (0–2)	1 (0.3–1.5)	1 (0.3–1.3)	NS
**Metastasis Size**						
IHC	95 (16%)	79 (16%)	7 (13%)	5 (16%)	4 (16%)	0.08
Micro	179 (31%)	154 (32%)	11 (24%)	11 (34%)	3 (32%)	
Macro	300 (52%)	247 (51%)	27 (60%)	16 (50%)	10 (51%)	
**Metastatic non sentinel axillary lymph node**						
Positive	136 (24%)	114 (24%)	11 (24%)	10 (31%)	1 (6%)	NS
**Number of metastatic non sentinel axillary lymph node**						
0	435 (76%)	362 (76%)	35 (75%)	22 (69%)	16 (94%)	NS
1	78 (14%)	65 (14%)	10 (22%)	3 (9%)	0	
2	25 (4%)	21 (4%)	0	3 (9%)	1 (6%)	
3	15 (3%)	12 (2%)	1 (2%)	2 (6%)	0	
>3	18 (4%)	16 (3%)	0	2 (6%)	0	

**Table 3:** Dataset 1. Dataset 2. Dataset 1 & 2.

MSKCC Discrimination by Molecular Subtypes. Dataset 1						
	All Samples	ERpos HER2neg	ERneg HER2neg	ERpos HER2pos	ERneg HER2pos	HER2pos
**Samples**	654	573 (88%)	22 (3%)	32 (5%)	27 (4%)	59 (9%)
**AUC (CI)**	0.73 (0.68–0.77)	0.73 (0.69–0.78)	0.95 (0.83–1)	O.66 (0.45–0.88)	0.66 (0.44–0.87)	0.67 0.52–0.82
**MSKCC Calibration by Molecular Subtypes. Dataset 1**						
**U:p**	0.001	0.001	0.01	0.39	0.59	0.24
**Emax**	0.13	0.14	0.45	0.13	0.16	0.14
**Eavg**	0.06	0.06	0.17	0.14	0.09	0.08

MSKCC Discrimination by Molecular Subtypes. Dataset 2						
	All Samples	ERpos HER2neg	ERneg HER2neg	ERpos HER2pos	ERneg HER2pos	HER2pos
**Samples**	574	480 (84%)	45 (8%)	32 (6%)	17 (3%)	49 (9%)
**AUC (CI)**	0.71 (0.65–0.76)	0.71 (0.65–0.76)	0.64 (0.4–0.8)	0.76 (0.56–0.96)	1	0.71 (0.5–0.93)
**MSKCC Calibration by Molecular Subtypes. Dataset 2**						
**U:p**	1e-05	1e-04	0.5	0.7	5e-05	0.06
**Emax**	0.18	0.16	0.25	0.07	1	0.7
**Eavg**	0.07	0.07	0.1	0.06	0.3	0.2

MSKCC Discrimination by Molecular Subtypes. Dataset 1 and 2						
	All Samples	ERpos HER2neg	ERneg HER2neg	ERpos HER2pos	ERneg HER2pos	HER2pos
**Samples**	1228	1053 (86%)	67 (5%)	64 (5%)	44 (4%)	108 (9%)
**AUC (CI)**	0.72 (0.68–0.75)	0.72 (0.69–0.76)	0.69 (0.51–0.89)	0.71 (0.56–0.85)	0.61 (0.4–0.8)	0.68 (0.57–0.8)
**MSKCC Calibration by Molecular Subtypes. Dataset 1 and 2**						
**U:p**	2.4e-8	1.8e-7	0.36	0.7	0.05	0.1
**Emax**	1.5e–1	1.5e–1	0.16	0.08	0.4	0.19
**Eavg**	6.9e–2	6.8e–2	0.11	0.09	0.1	0.09

MSKCC Nomogram discrimination and calibration. AUC, Area Under Curve. CI, Confidence Interval. U:p, Unreliability test p value. Emax, Maximal Error. Eavg, Average Errors.

The aim of our study was to analyze how molecular subtypes classification interacts with the non-sentinel node status of breast cancer patients with metastatic sentinel lymph node. As the two predictors described above were built on large datasets and validated thoroughly, they both hypothetically accurately picture the subtle interactions existing between the primary tumour clinical and pathological features, the metastatic sentinel nodes and the corresponding non-sentinel node status. We applied the Tenon Score and the MSKCC nomogram to two large independent datasets of 656 and 574 breast cancer patients with metastatic sentinel nodes and full assessment of ER and *HER2* status.

**Figure 1:**
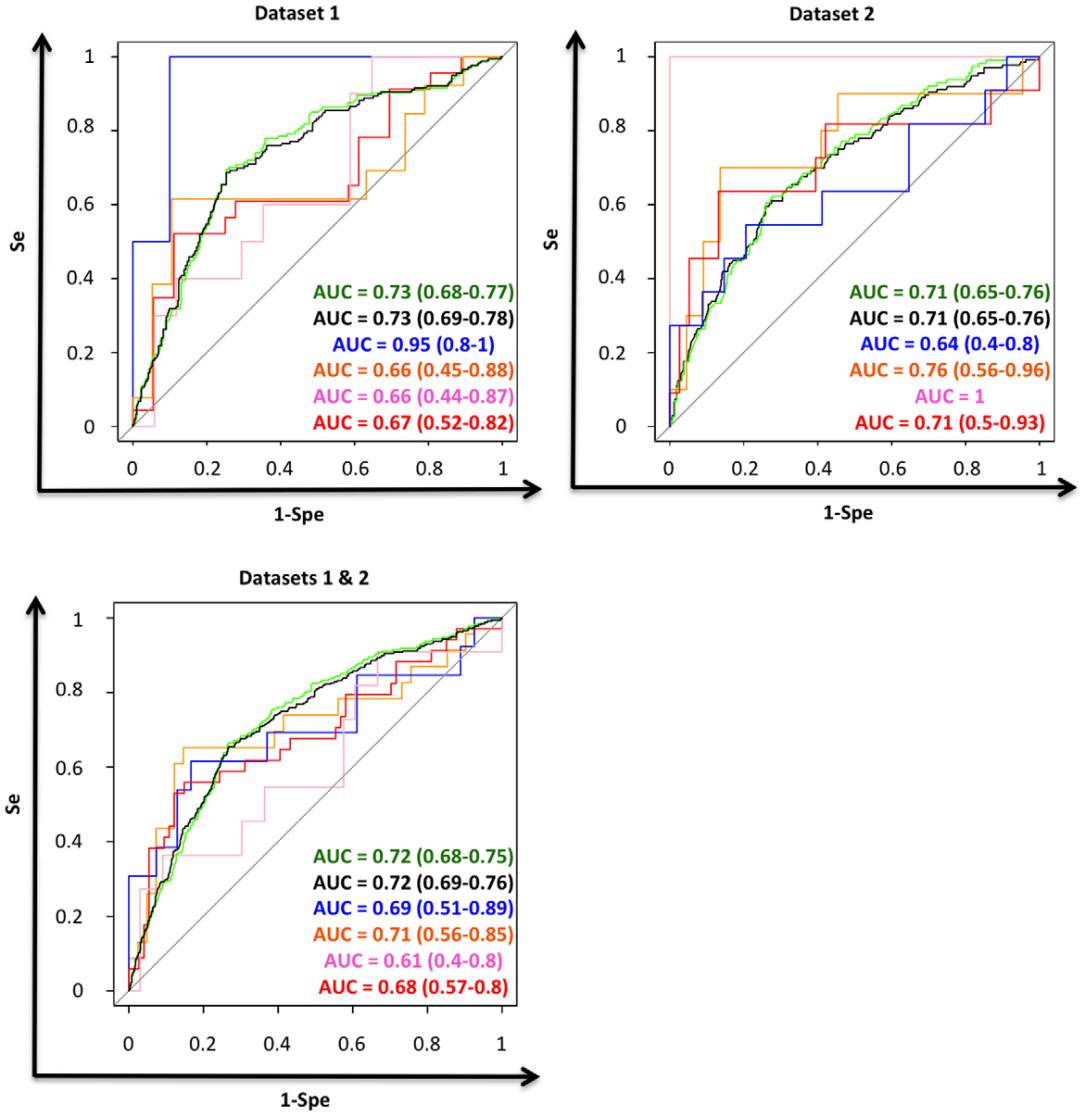
Discrimination of the MSKCC Nomogram for each Immuno-Phenotype Subtypes. Top left: Dataset 1. Discrimination of the MSKCC Nomogram for each immuno-phenotype subtypes. Green = Whole dataset. Black = ER positive *HER2* negative subgroup. Blue = ER negative *HER2* negative subgroup. Orange = ER positive *HER2* positive subgroup. Pink = ER negative *HER2* positive subgroup. Red = *HER2* positive subgroup. Top right: Dataset 2. Discrimination of the MSKCC Nomogram for each immuno-phenotype subtypes. Green = Whole dataset. Black = ER positive *HER2* negative subgroup. Blue = ER negative *HER2* negative subgroup. Orange = ER positive *HER2* positive subgroup. Pink = ER negative *HER2* positive subgroup. Red = *HER2* positive subgroup. Bottom Left: Dataset 1 & 2. Discrimination of the MSKCC Nomogram for each immuno-phenotype subtypes. Green = Whole dataset. Black = ER positive *HER2* negative subgroup. Blue = ER negative *HER2* negative subgroup. Orange = ER positive *HER2* positive subgroup. Pink = ER negative *HER2* positive subgroup. Red = *HER2* positive subgroup.

## Materials and Methods

### Patients

Our first dataset consisted of 656 early-stage breast cancer patients treated between 2005 and 2009 and identified through the Institut Curie (Paris) prospective breast cancer database. Our second dataset consisted of 574 early-stage breast cancer patients treated between 2005 and 20011 and identified through the Institut Curie (Saint-Cloud) prospective breast cancer database. The main inclusion criterion were patients with an infiltrating breast carcinoma <30 mm based on clinical and radiological features, normal physical examination of the axilla, treated at first by conservative surgery plus a sentinel node (SN) biopsy. The procedure was performed with patent blue, radioisotope or a combination, as previously described, in line with French recommendations. SN biopsy was performed as previously described [7]. Axillary lymph node dissection was performed during the same procedure when the SN was positive by imprint cytology or frozen section. A second operation was performed when either hematoxylin-eosin staining or immunohistochemistry revealed tumour cells in the SN postoperatively, including isolated tumour cells. Pathologic SN examination methods were as reported previously [7]. Patients receiving a neoadjuvant treatment (chemotherapy, hormone-therapy or radiotherapy) or with a locoregional recurrence were systematically excluded from the study. The clinical data (age at diagnosis, treatment protocols) were extracted from the both Institut Curie (Paris and Saint-Cloud) prospective breast cancer database.

**Figure 2:**
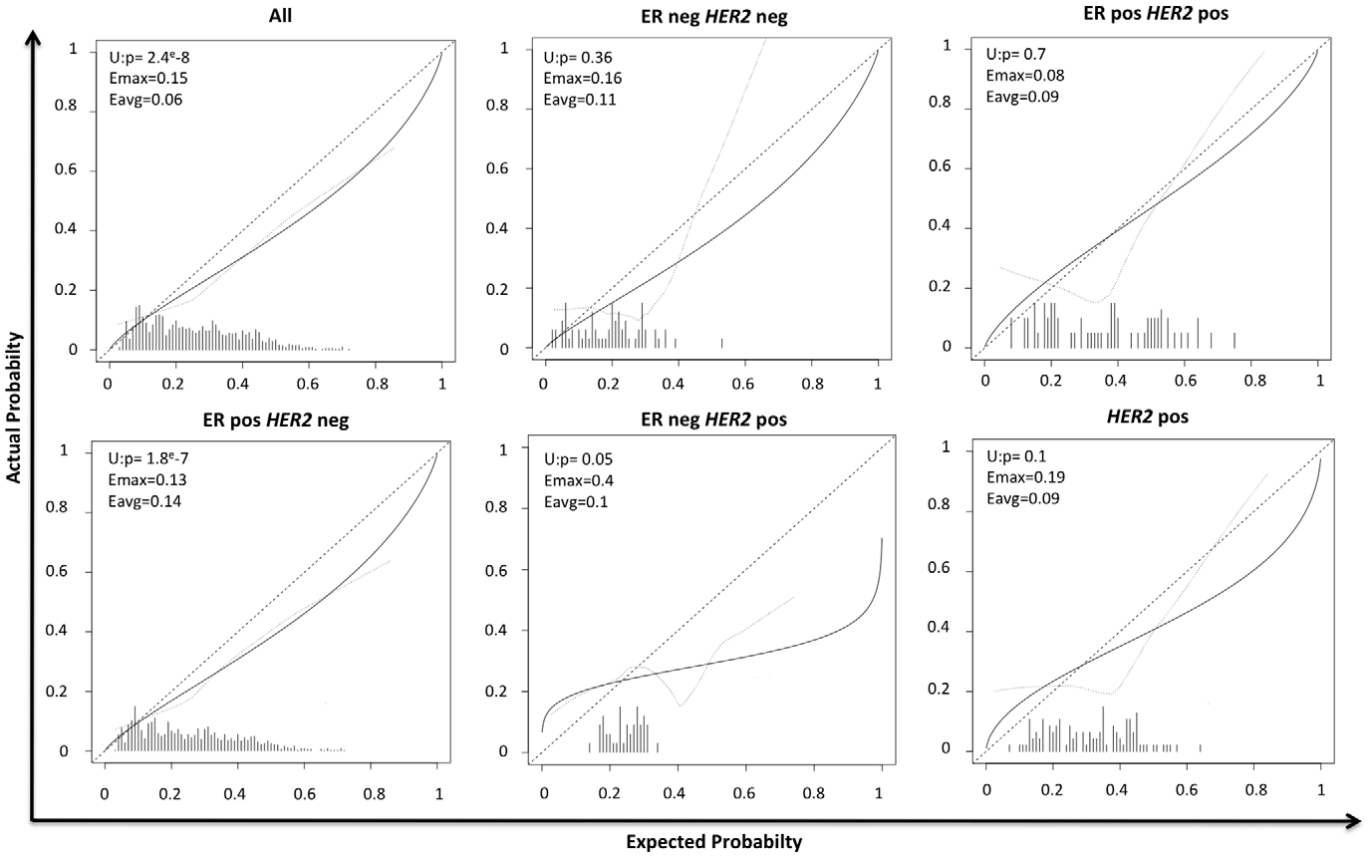
Calibration of the MSKCC Nomogram for each immuno-phenotype subtypes. Dataset 1 & 2.

### Tumour samples

The following histological features were retrieved: tumour type, tumour size, Lympho Vascular Invasion, Estrogen Receptor status, *HER2* status, number of metastatic sentinel nodes (IHC, micro, macro), number of sentinel nodes, size of sentinel node metastasis, histologic detection of SN metastasis, number of non-sentinel nodes removed, number of metastatic non-sentinel node. Estrogen Receptor (ER) was determined as follow. After rehydration and antigenic retrieval in citrate buffer (10 mM, pH 6.1), the tissue sections were stained for estrogen receptor (clone 6F11, Novocastra, 1/200). Revelation of staining was performed using the Vectastain Elite ABC peroxidase mouse IgG kit (Vector Burlingame, CA) and diaminobenzidine (Dako A/S, Glostrup, Denmark) as chromogen. Positive and negative controls were included in each slide run. Cases were considered positive for ER according to standardized guidelines using ≥10% of positive nuclei per carcinomatous duct. The determination of *HER2* over-expression status was determined according to the American Society of Clinical Oncology (ASCO) guidelines [24].

**Figure 3:**
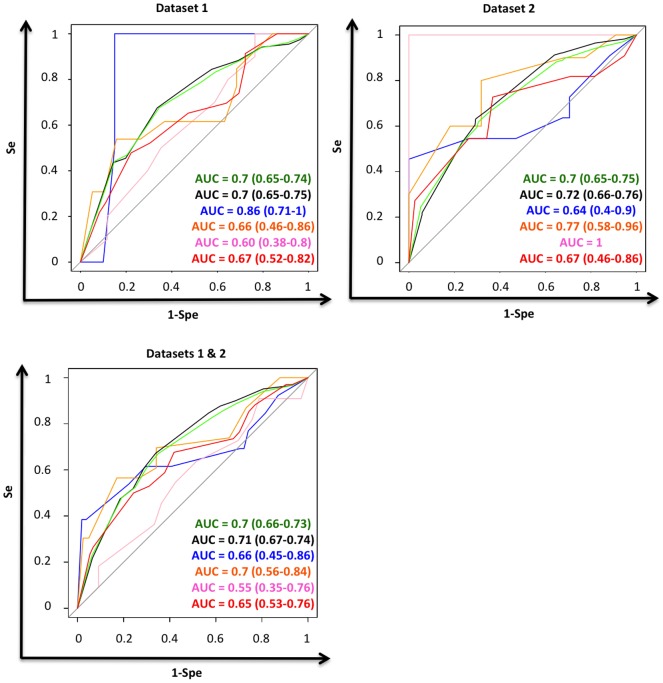
Discrimination of the Tenon score for each Immuno-Phenotype Subtypes. Top left: Dataset 1. Discrimination of the Tenon Score for each immune-phenotype subtypes. Green = Whole dataset. Black = ER positive *HER2* negative subgroup. Blue = ER negative *HER2* negative subgroup. Orange = ER positive *HER2* positive subgroup. Pink = ER negative *HER2* positive subgroup. Red = *HER2* positive subgroup. Top right: Dataset 2. Discrimination of the Tenon Score for each immune-phenotype subtypes. Green = Whole dataset. Black = ER positive *HER2* negative subgroup. Blue = ER negative *HER2* negative subgroup. Orange = ER positive *HER2* positive subgroup. Pink = ER negative *HER2* positive subgroup. Red = *HER2* positive subgroup. Bottom Left: Dataset 1 & 2. Discrimination of the Tenon Score for each immune-phenotype subtypes. Green = Whole dataset. Black = ER positive *HER2* negative subgroup. Blue = ER negative *HER2* negative subgroup. Orange = ER positive *HER2* positive subgroup. Pink = ER negative *HER2* positive subgroup. Red = *HER2* positive subgroup.

**Table 4:** Dataset 1. Dataset 2. Dataset 1 & 2.

Tenon Score Discrimination by Molecular Subtypes. Dataset 1						
	All Samples	ERpos HER2neg	ERneg HER2neg	ERpos HER2pos	ERneg HER2pos	HER2pos
**Samples**	654	573 (88%)	22 (3%)	32 (5%)	27 (4%)	59 (9%)
**AUC (CI)**	0.70 (0.65–0.74)	0.7 (0.65–0.75)	0.86 (0.71–1)	0.66 (0.46–0.86)	0.60 (0.38–0.8)	0.64 (0.49–0.78)

Tenon Score Discrimination by Molecular Subtypes. Dataset 2						
	All Samples	ERpos HER2neg	ERneg HER2neg	ERpos HER2pos	ERneg HER2pos	HER2pos
**Samples**	574	480 (84%)	45 (8%)	32 (6%)	17 (3%)	49 (9%)
**AUC (CI)**	0.7 (0.65–0.75)	0.72 (0.66–0.76)	0.64 (0.4–0.88)	0.77 (0.58–0.96)	1	0.67 (0.46–0.86)

Tenon Score Discrimination by Molecular Subtypes. Dataset 1 and 2						
	All Samples	ERpos HER2neg	ERneg HER2neg	ERpos HER2pos	ERneg HER2pos	HER2pos
**Samples**	1228	1053 (86%)	67 (5%)	64 (5%)	44 (4%)	108 (9%)
**AUC (CI)**	0.7 (0.66–0.73)	0.71 (0.67–0.74)	0.66 (0.45–0.86)	0.70 (0.56–0.84)	0.55 (0.35–0.76)	0.65 (0.53–0.76)

Tenon Score discrimination. AUC, Area Under Curve. CI, Confidence Interval.

The SLN histopathological assessment protocol has been published by Fréneaux et al [7]. SLN samples were serially sectioned and stained with HE. Negative HE cases were then analyzed by serial sectioning with IHC. Positive sentinel nodes were classified into two groups according to the size of the metastasis: macrometastasis (>2 mm) and micrometastasis (< = 2 mm) detected either by HE staining or by cytokeratin IHC.

**Figure 4:**
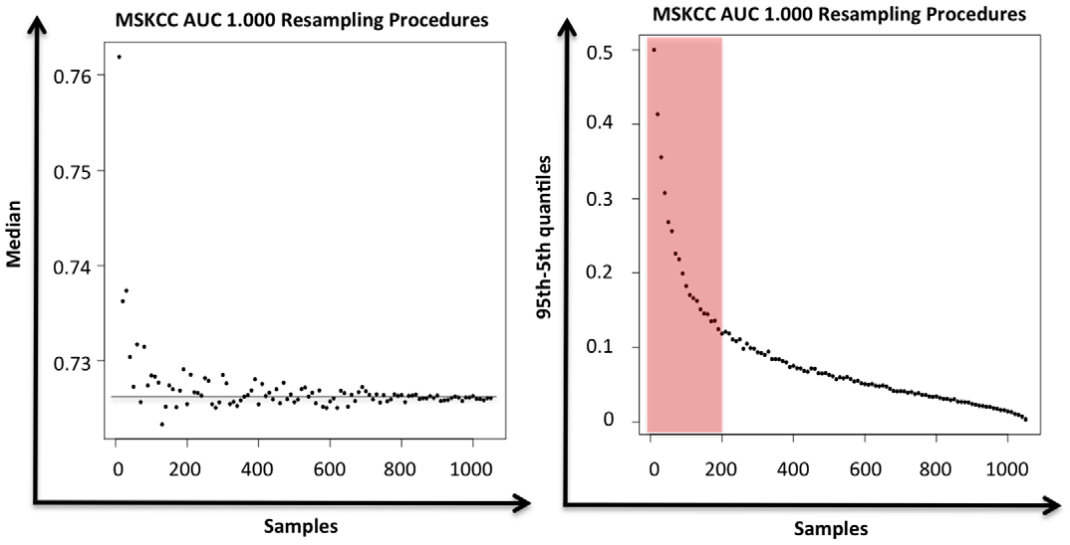
Impact of sample size on the MSKCC nomogram performance. Left: 10.000 resampling procedures of 10 to 1050 (increment series by 10 samples) ER positive *HER2* negative breast cancer samples. MSKCC AUC median value for each re-sampling categories. Right: 10.000 resampling procedures of 10 to 1050 (increment series by 10 samples) ER positive *HER2* negative breast cancer samples. MSKCC AUC 95^th^ percentile minus MSKCC AUC 5^th^ percentile value for each re-sampling categories.

### Statistical model

Baseline characteristics were compared between groups using Chi-square or Fisher's exact tests for categorical variables and Student's t-tests for continuous variables. The Tenon Score was calculated as previously described (Histologic tumour size: 0 point when < = 10 mm, 1.5 point when 0 to 20 mm, 3 points when>20 mm. Size of sentinel node metastasis : 2 points when macrometastasis, 0 if not. Ratio of positive sentinel nodes to all removed sentinel nodes : 0 point when < = 0.5, 1 point when 0.5–1, 2 points when  = 1. Score 0 to 7. Threshold < = 3.5) [1]. The MSKCC nomogram was obtained from a dedicated website (http://nomograms.mskcc.org/Breast/BreastAdditionalNonSLNMetastasesPage.aspx). The probability of non sentinel node metastases was calculated using the following variables: tumour type, tumour grade, pathological tumour size, number of positive sentinel lymph nodes, sentinel lymph node method of detection, number of negative sentinel lymph nodes, lymphovascular invasion, multifocality, estrogen receptor status.

The model performance was quantified with respect to discrimination and calibration. Discrimination (i.e., whether the relative ranking of individual predictions is in the correct order) was quantified with the area under the receiver operating characteristic curve. Calibration (i.e., agreement between observed outcome frequencies and predicted probabilities) was studied with graphical representations of the relationship between the observed outcome frequencies and the predicted probabilities (calibration curves): the grouped proportions versus mean predicted probability in groups defined by deciles and the logistic calibration were represented. Well-calibrated models have an intercept alpha = 0 and a slope Beta = 1. Therefore, a sensible measure of calibration is a likelihood ratio statistic testing the null hypothesis that α = 0 and β = 1. The statistic has a χ2 distribution with 2 df (unreliability [U] statistic). We also evaluated average errors [Eaver] and maximal errors [E max] between predictions and observations obtained from a calibration curve. Calibration is not adequate to evaluate a score that are intended to give a positive or negative result.

The analyses were performed using R software (http://cran.r-project.org). The breast cancer study group of the Institut Curie (Paris and Saint-Cloud) approved the study.

### Ethics Statement

The registration of patients of the Institut Curie (Paris and Saint-Cloud) in this cohort received a favorable agreement of the french National Committee on Computers and Liberties (CNIL, Commission nationale de l'informatique et des libertés). Patients gave informed written consent prior to be registered in the cohort. The study was approved by the breast cancer study group and the comity of clinical research study of the Institut Curie (Paris and Saint-Cloud).

## Results

Table 1 summarizes the clinical and pathological features of 656 early-stage breast cancer patients treated initially by conservative surgery and sentinel node procedure, between 2005 and 2009 at Institut Curie, Paris. 574 (88%) tumours were classified as ER positive *HER2* negative, 22 (3%) were ER negative *HER2* negative, 32 (5%) were ER positive *HER2* positive and 28 (4%) were ER negative *HER2* positive. We identified significant differences between these 4 categories in terms of histological type (14% were lobular carcinoma in ER positive *HER2* negatives subgroup, 13% were “other type i.e medullary carcinoma” in ER negative *HER2* negative subgroup, p = 9e–05), lympho-vascular space involvement (34%, 36%, 47%, 60%, p = 0.02), multifocality (19%, 0%, 31%, 29%, p = 0.02), and percentage of non-sentinel axillary lymph node metastasis (27%, 9%, 41%, 39%, p = 0.03). Table 2 summarizes the clinical and pathological features of 574 early-stage breast cancer patients treated initially by conservative surgery and sentinel node procedure, between 2005 and 2011 at Institut Curie, Saint-Cloud. 480 (84%) tumours were classified as ER positive *HER2* negative, 45 (8%) were ER negative *HER2* negative, 32 (6%) were ER positive *HER2* positive and 17 (3%) were ER negative *HER2* positive.

### Performance of the Memorial Sloan-Kettering Cancer Center nomogram. Dataset 1 (Institut Curie, Paris)

When applied to the whole population the MSKCC Nomogram has an AUC of 0.73 (0.68–0.77) (Table 3). The probability of non-sentinel node metastatic involvement was significantly under-estimated by the MSKCC nomogram. We performed a subgroup analysis based on the immune-phenotype molecular subtypes classification and showed an unbalance performance. The ER positive *HER2* negative subgroup showed expected performance (AUC = 0.73 (0.69–0.78)). The probability of non-sentinel node metastatic involvement was significantly under-estimated by the MSKCC nomogram. The ER negative *HER2* negative subgroup had the highest AUC (0.95 [0.83–1]) followed by the ER positive *HER2* negative subgroup (0.73 [0.69–0.78]). The MSKCC nomogram was unable to discriminate between non-sentinel status (metastasis vs no metastasis) in the *HER2* subgroups (ER positive *HER2* positive, ER negative *HER2* positive), whatever the ER status was. We had too few samples in the ER negative *HER2* negative subgroup to interpret its calibration curve. Concerning the *HER2* positive subgroups, as the discrimination is non-significant, interpretation of the calibration curves remains uncertain. The average and maximal errors were relatively high.

### Performance of the Memorial Sloan-Kettering Cancer Center nomogram. Dataset 2 (Institut Curie, Saint-Cloud)

When applied to the whole population the MSKCC Nomogram has an AUC of 0.71 (0.65–0.76) (Table 3. Figure 1). We performed a subgroup analysis based on the immune-phenotype molecular subtypes classification and showed an unbalance performance. The ER positive *HER2* negative subgroup showed expected performance (AUC = 0.71 (0.65–0.76)). The probability of non-sentinel node metastatic involvement was significantly under-estimated by the MSKCC nomogram. The ER negative *HER2* positive subgroup had the highest AUC (AUC = 1) followed by the ER positive *HER2* positive subgroup (0.76 [0.56–0.96]). The MSKCC nomogram was unable to discriminate between non-sentinel status (metastasis vs no metastasis) in the ER negative *HER2* negative subgroup. Probability of non-sentinel node metastatic involvement in ER positive *HER2* negative subgroup was significantly under-estimated by the MSKCC nomogram. Concerning the *ER* negative *HER2* negative and the *HER2* positive subgroups, as the discrimination is non-significant, interpretation of the calibration curves remains uncertain. The average and maximal errors were relatively high.

### Performance of the Memorial Sloan-Kettering Cancer Center nomogram. Combination of both datasets. (Institut Curie, Paris, Saint-Cloud)

The performance in terms of calibration and discrimination was similar in both datasets in the ER positive *HER2* negative subgroup and was contradictory in other molecular subgroups (ER negative *HER2* negative, ER negative *HER2* positive, ER positive *HER2* positive, *HER2* positive). A lack of power was the main hypothesis to explain these results. We repeated the same analysis after merging the two datasets. A total of 1228 samples were analyzed: 1053 samples were ER positive *HER2* negative (86%), 67 samples were ER negative *HER2* negative (5%), 64 samples were ER positive *HER2* positive (5%) and 44 samples were ER negative *HER2* positive (4%). When applied to the whole population the MSKCC nomogram has an AUC of 0.72 (0.68–0.75) (Table 3). We performed a subgroup analysis based on the immune-phenotype molecular subtypes classification. The ER positive *HER2* negative subgroup showed expected performance (AUC = 0.72 (0.69–0.76)). Probability of non-sentinel node metastatic involvement in ER positive *HER2* negative subgroup was significantly under-estimated by the MSKCC nomogram (Figure 2). We showed a shift to the ER positive *HER2* negative subgroup MSKCC nomogram performance (discrimination and calibration) in the ER negative *HER2* negative, ER negative *HER2* positive, and *HER2* positive subgroups. We remained unable to discriminate between non-sentinel status (metastasis vs no metastasis) in the ER negative *HER2* positive subgroup. This specific subgroup was the smallest one with 44 samples.

#### Tenon Score

Same results were obtained with the Tenon score (Table 4 and Figure 3). Tenon score as a score could not be analyzed in terms of calibration.

#### Small sample size issue

To resolve this interrogation we performed an iterative sampling (10.000) of 10 to 1050 samples (series increment by 10 samples i.e. 10, 20, 30…) out of the 1053 ER positive *HER2* negative samples and quantified with the area under the receiver operating characteristic curve the discrimination performance of the MSKCC predictor (Figure 4). It showed a great variability of the predictor when applied to small dataset with less than 200 samples. The AUC median value for each sampling size was relatively stable even in very small datasets (below 100 samples). Conversely the difference between the AUC 95^th^ and 5^th^ percentiles was dramatically decreasing from 10 to 200 sampling size.

## Discussion

The aim of our work was to decipher the relation between the molecular subtype classification as defined by a combination of *ER* and *HER2* status and the probability of a positive non-sentinel node biopsy of breast cancer patients with metastatic sentinel lymph node. In order to validate this hypothesis we tested the performance of the Tenon Score (TS) and MSKCC nomogram (MKCC) designed to predict non-sentinel lymph node status in patients with sentinel node metastasis [1], [20]. These scores were built and validated thoroughly on large independent datasets [4], [19]. We applied these predictors to the whole population and performed a subgroup analysis after stratification of the whole dataset based on the immune-phenotype molecular subtypes classification as defined by a combination of ER and *HER2* immuno-histochemistry status. The analysis was performed with two large independent datasets from our institution. We showed that the performance of the two predictors in terms of discrimination was high in ER positive *HER2* negative subgroup in both datasets. Probability of non-sentinel node metastatic involvement was slightly under-estimated by the MSKCC nomogram in both datasets (Tenon score as a score could not be analyzed in terms of calibration).

We showed contradictory results in other molecular subgroups in both datasets. Due to the small sample size issue of these specific subgroups we performed the same analysis after merging the two datasets. We showed a shift to the ER positive *HER2* negative subgroup MSKCC nomogram performance (discrimination and calibration) and Tenon score discrimination in the ER negative *HER2* negative, ER negative *HER2* positive, and *HER2* positive subgroups. A resampling procedure performed with the large ER positive *HER2* negative dataset (1053 samples) accurately pictured the performance variability of these predictors when the sample size is below 200.

Conversely to the sentinel node status, we showed that non-sentinel node metastasis of breast cancer patients with metastatic sentinel node is potentially independent of the underlying biology as determined by the molecular immune-phenotype classification. Several authors have recently underscored a significant relation between the molecular subtypes classification and the axillary status of breast cancer patients [5], [9]–[12], [14], [18], [23]. We previously published a multivariate logistic regression model to determine the probability of having a positive sentinel node biopsy combining the tumour size, lympho-vascular invasion, age at diagnosis and the molecular subtypes classification [16]. Furthermore we identified for each molecular subtype a specific correlation pattern between the tumour size and the probability of a positive sentinel node biopsy. The ER negative *HER2* negative breast cancer subgroup nodal status was almost independent from the tumour size with a relative constant trend of axillary metastases around 20%. Conversely the ER positive or *HER2* positive breast cancer subgroups showed a strong and almost linear correlation between the tumour size and the percentage of axillary metastasis. Lu et al identified a similar multivariate model to predict lymph node metastases that included tumour size, lympho vascular invasion and tumour subtypes and modified Bloom and Richardson grade [12]. These evidences sustained the idea that nodal status is a potential signature of the intrinsic biological properties of a primary tumour.

We showed here that a predictor like the Tenon Score or the MSKCC nomogram built on a heterogeneous population of breast cancer (containing diverse histology and molecular subtypes) is performing equally on the different molecular subgroups analyzed. Our present study re-enforce the idea that performing subgroup analysis of predictors like the Tenon Score or the MSKCC nomogram within less than 200 samples subgroup is at major risk of misleading conclusions. To draw definitive conclusion concerning the potential independency of the molecular subtypes classification and the non sentinel node status in breast carcinoma with metastatic sentinel lymph node, we intend to analyze a large series of sentinel node positive *HER2* positive and sentinel node positive ER negative *HER2* negative breast cancer samples.
